# Deep phylogeny, ancestral groups and the four ages of life

**DOI:** 10.1098/rstb.2009.0161

**Published:** 2010-01-12

**Authors:** Thomas Cavalier-Smith

**Affiliations:** Department of Zoology, University of Oxford, South Parks Road, Oxford OX1 3PS, UK

**Keywords:** bacteria, protozoa, symbiogenesis, kingdoms of life, lateral gene transfer, classification

## Abstract

Organismal phylogeny depends on cell division, stasis, mutational divergence, cell mergers (by sex or symbiogenesis), lateral gene transfer and death. The tree of life is a useful metaphor for organismal genealogical history provided we recognize that branches sometimes fuse. Hennigian cladistics emphasizes only lineage splitting, ignoring most other major phylogenetic processes. Though methodologically useful it has been conceptually confusing and harmed taxonomy, especially in mistakenly opposing ancestral (paraphyletic) taxa. The history of life involved about 10 really major innovations in cell structure. In membrane topology, there were five successive kinds of cell: (i) negibacteria, with two bounding membranes, (ii) unibacteria, with one bounding and no internal membranes, (iii) eukaryotes with endomembranes and mitochondria, (iv) plants with chloroplasts and (v) finally, chromists with plastids inside the rough endoplasmic reticulum. Membrane chemistry divides negibacteria into the more advanced Glycobacteria (e.g. Cyanobacteria and Proteobacteria) with outer membrane lipolysaccharide and primitive Eobacteria without lipopolysaccharide (deserving intenser study). It also divides unibacteria into posibacteria, ancestors of eukaryotes, and archaebacteria—the sisters (not ancestors) of eukaryotes and the youngest bacterial phylum. Anaerobic eobacteria, oxygenic cyanobacteria, desiccation-resistant posibacteria and finally neomura (eukaryotes plus archaebacteria) successively transformed Earth. Accidents and organizational constraints are as important as adaptiveness in body plan evolution.

## 1. Introduction

The nature of the deepest branches in the evolutionary tree and the last common ancestor of all life are key questions in biology having wide ramifications. Currently, we are in the early stages of a paradigm shift in which the prevailing view on these matters should be replaced by a sounder one. This review summarizes recent insights into bacterial and protozoan large-scale evolution and the tree of life for non-specialists and argues that much more intense research into the little-known phylum Chlorobacteria is needed for better understanding the nature of our last common ancestor. To avoid burdening you with excessive detail, I do this rather briefly in the second half of this essay, giving references to specialist literature for those wanting more detail or evidence for my conclusions.

The first half is a broader historical/philosophical discussion of the contrast between ancestral and derived groups and how taxonomists should handle them. The past three decades have seen a dramatic increase in the use of DNA sequences for reconstructing phylogeny and a parallel shift in emphasis from evolutionary taxonomy (Mayr 1974) towards Hennig's (1966) ‘phylogenetic systematics’, often accompanied by much controversy. Great advances in knowledge and understanding of organismal history have been made, but some fashions, attitudes and dogmas have spread more widely and dominated other viewpoints more than their scientific merits justify. The significance of the stasis of ancestral body plans over billenia and the non-uniformity of evolutionary modes and rates is insufficiently appreciated. Much discussion has been among students of recently derived branches of the tree (Hennig insects; Mayr birds) or among those whose focus is biochemistry or computer algorithms, rather than organisms and the needs and principles of taxonomy. I offer the perspective of a biologist especially interested in unicellular organisms, ancestral groups and in explaining the major transitions of life, perhaps more conscious than most of flaws in some aspects of recent phylogenetic fashions.

Soon after it was founded, the Royal Society published *Micrographia* in which Hooke (1664) applied the word cell for the first time to the walled units of dead plant tissues that he first depicted. However, the modern concept of cells as living units that multiply by division grew up only in the mid nineteenth century simultaneously with that of evolution by variation and selection. Weismann (1889) made the first synthesis of cell biology and evolution, in which cell lineages were seen as the physical basis for inheritance and evolution, but his emphasis was all on nucleated cells, as was Wallace's (1911) claim that only a creative mind could have made them. Understanding the big picture of organismal history requires more attention than hitherto to the main features of the evolution of sexless bacterial cells which exclusively dominated the biosphere for three-quarters of its history. More than all eukaryotes together, bacteria still largely manipulate biogeochemical cycles and global climate.

Though I shall not dwell on it, another limitation of Weismann's synthesis has become apparent in the past two decades. Superimposed on the vertical inheritance of cell lineages that Weismann recognized is the horizontal transfer of individual genes or small clusters of genes among organisms of separate cell lineages, which can affect the evolution of extremely distantly related organisms. In bacteria, such lateral gene transfer (LGT) occurs mainly by viruses, plasmids or the uptake of naked DNA from dead cells. In eukaryotes, feeding by phagocytosis followed by inefficient digestion of prey DNA and its accidental incorporation into nuclear chromosomes is probably how protozoa most often get foreign genes (Doolittle 1998). Although LGT of DNA independently of cell lineages is evolutionarily important, especially among bacteria and protozoa (see Cavalier-Smith submitted *a*), it seems to have played no role in the evolution of the major cellular body plans that I focus on here.

## 2. Early perceptions of cell lineages and the unity of life

Shall we conjecture that one and the same kind of living filaments is and has been the cause of all organic life?

(Darwin 1794, p. 507)

The nucleated vesicle, the fundamental form of all organization, we must regard as the meeting point between the inorganic and the organic—the end of the mineral and beginning of the animal and vegetable kingdoms … We have already seen that this nucleated vesicle is itself a type of mature and independent being in the infusory animalcule [now called Protozoa, following von Siebold (1845)] … The first step in the creation of life upon this planet was a chemico-electric operation, by which simple germinal vesicles were produced … What were the next steps? … an advance under favour of peculiar conditions, from the simplest forms of being to the next most complicated, and this through the ordinary process of generation.

(Chambers 1844, pp. 204–205)

Robert Chambers, the author of the first English book on evolution, and his brother were born with six digits on each limb. Probably, this alerted him to inherited mutations being the primary cause of evolution by descent with modification, an idea adumbrated by de Maupertuis (1745), who pioneered the mathematical genetics of polydactyly (de Maupertuis 1751). Understandably, Chambers wrote anonymously, like de Maupertuis and De Maillet (1748)—the first modern advocate of evolution and a common ancestry for radically different animals, who took the further precaution of publishing posthumously; then the dictum was ‘publish and perish’. Chambers wrote well after the Pope banned *Zoonomia* by Charles Darwin's grandfather Erasmus, who first correctly suggested that all organisms evolved by modifying a single microbial common ancestor (Darwin 1794), but during the British backlash against progressive ideas, especially foreign French ones like those of de Maupertuis, De Maillet and Lamarck (1809).

Lamarck in French and Chambers in English first proposed phylogenetic trees for real organisms. Darwin (1859) scrupulously avoided doing that. Unmerited ridicule as they suffered would have jeopardized his threefold mission: demonstrating the fact of evolution; showing how the struggle for existence explains adaptation; and attempting to explain evolutionary change (transformation) by genetic variation and the differential multiplication of genotypes. When emphasizing how common ancestry plus divergence into novel phenotypes explain the striking patterns of similarity and differences that enable us to classify organisms into successively nested taxa within higher taxa, Darwin cleverly used an abstract tree immune to ridicule or phylogenetic error. He correctly argued that the body plan shared by members of a phylum was present in their last common ancestor and has been stably inherited generation after generation, with no fundamental change for hundreds of millions of years. Proceeding down the hierarchy of categories through class, order, family, genus and species, each successive subordinate group differs from its closest relatives in characters of decreasing long-term stability (Lamarck 1809).

Chambers wrote shortly after Meyen (1839), Dujardin (1841) and Barry (1843) unified biology by showing that protozoa are single nucleated cells homologous with those forming animal and plant bodies and that continuous cell lineages are the physical basis of life. (The more famous Schleiden (1838) and Schwann (1839) whom text books call ‘the’ authors of ‘cell theory’ did not realize this.) But a century elapsed before electron microscopy clarified the fundamental distinction between bacteria and nucleated (eukaryotic) cells. We now know that life did not begin with protozoa having cells like ours, as Chambers thought, but with much simpler bacterial (prokaryotic) cells. On the most conservative estimate, nucleated cells evolved only approximately 800–850 Myr ago (Cavalier-Smith 2002*b*, 2006*a*). For the first 2.6 billion years, only bacteria inhabited the world. Microscopically simple, but structurally exceedingly complex in their atomic arrangements, their fantastic diversity and biochemical ingenuity is mediated by thousands of intricate macromolecular machines whose three-dimensional structure and interactions are revealed only by X-ray crystallography. As noted above, bacterial evolution has depended only on the evolutionary divergence of cell lineages plus the horizontal transfer of DNA. The much later origin of protozoa with their sexual gamete fusion and predation by phagocytosis (Cavalier-Smith 2009*b*) made the merger of cell lineages a novel factor in evolution and population genetics (commonly by sex and extremely rarely by symbiogenesis of foreign engulfed cells to form organelles, like mitochondria and chloroplasts; Cavalier-Smith 2000, 2006*b*).

## 3. Common ancestry, stasis and divergence in the history of life

Explaining stasis is as important as explaining change. Darwin correctly divined the key role of selection in promoting adaptation and in channelling the historical divergence of related members of a taxon. But he did not sufficiently realize its importance also in ensuring stable inheritance over billions of generations of ancestral body plans, though unlike Chambers he refused to attribute such plans ultimately to a creative mind. Only after twentieth-century understanding of the physical inevitability of mutations affecting every single nucleotide of a genome could we appreciate the fundamental significance of purifying and stabilizing selection in preserving body plans over billenia (Schmalhausen 1949). Inheritance alone is too imperfect to achieve this. About half the nucleotides in ribosomal RNA (rRNA) molecules have an identical sequence in every bacterium, animal, plant and fungus, despite every nucleotide regularly mutating, some in every generation in every species. Since you started reading this paper, at least one cell of your body will have one or more new mutations in regions of rDNA where the ancestral sequence in the last common ancestor of all life has never actually been supplanted by evolution over 3.5 billion years. The same applies to hundreds of other genes essential for life. Stasis stems from the lethality (or dramatically lower fertility) of such variants (purifying selection) and is not inherent to the genetic material. Without death, life could not persist. Contrary to what Darwin thought, and many creationists still do, the problem is less to explain how genetic variation occurs, than to understand why some organismal properties never change while others frequently do. Differential reproductive success (anthropomorphically ‘natural selection’) biases genotypes of successive generations subjected to a perpetual, physically inevitable, barrage of mutations in every part of the genome. This beautifully explains both long-term stasis and radical organismal transformation. Both stasis and change are needed to explain the patterns of similarity and difference that enable hierarchical Linnean classification.

## 4. The kingdoms and tree of life

Except for Lamarck's and Chambers’ ridiculed attempts and Goldfuss (1820), who introduced the name Protozoa (first in 1817) for the microscopic Infusoria that Lamarck put at the base of the animal kingdom, tracing the actual history of life in detail and explaining the origins of specific novel groups of organisms were begun in a bold and detailed way only by Darwin's contemporary and admirer Haeckel (1866). Haeckel coined the word phylogeny for the evolutionary history of a group, to contrast it with the development of an individual organism within one generation. Even Haeckel was ridiculed by some of my Cambridge zoology teachers, such was the antipathy to phylogeny among mechanistic biologists. Undiscriminating critics who attribute to him nasty social views he did not hold also unfairly denigrate his scientific genius. Haeckel made the important distinction between a group that shared ancestral characters because of their single (monophyletic) origin prior to their common ancestor (e.g. vertebrates whose last common ancestor already had a skull and vertebral column and many other features) and groupings of organisms (e.g. ‘flying animals’ or ‘parasitic plants’) that share characters that evolved several times independently, i.e. are polyphyletic.

Prior to Haeckel, organisms were generally divided into just two kingdoms: animals and vegetables, even though Necker (1783) and others later made a third kingdom for fungi, and Owen (1858) had placed unicellular organisms such as bacteria, amoebae and diatoms in a separate kingdom, Protozoa. Haeckel (1866) divided the tree of life into three branches, kingdoms Animalia, Plantae and Protista, each of which he thought arose monophyletically from the primordial slime. His kingdom Protista was heterogeneous, including heterotrophic bacteria, diatoms, amoebae and sponges; later, he moved amoebae and sponges to the animal kingdom and supposed that the residual Protista and life itself were highly polyphyletic. His three-kingdom system did not catch on, as critics thought it somewhat arbitrary what he placed in each. Only in a very loose sense was it a precursor of modern multikingdom systems. He placed heterotrophic bacteria in Protista and cyanobacteria (blue-green algae) in Plantae and thus failed to appreciate the basic unity of prokaryotes (first clearly recognized, as Schizophyta, by Cohn (1875), who visited Darwin in 1876) and the fundamental difference between prokaryotes and eukaryotes. This became accepted only after electron microscopy showed their ultrastructural differences: notably the absence of nuclei, mitochondria, an endomembrane system and internal cytoskeleton in all prokaryotes—and their universal presence in eukaryotes, coupled with the totally different ultrastructure of bacterial flagella and the unrelated eukaryotic cilia/flagella and basic differences in chromosome organization and cell division machinery. Stanier was chiefly responsible for recognizing the prokaryote–eukaryote dichotomy as the most fundamental in the living world (Stanier & Van Niel 1962; Stanier *et al*. 1963). Recent misguided criticisms notwithstanding, this two-fold division reflects a profound evolutionary truth (Cavalier-Smith 1991*a*,*b*, 2006*a*, 2007*b*).

Acceptance of prokaryotes as a distinct kingdom followed the influential paper by Whittaker (1969) who called it Monera, based on seminal work by Copeland (1956). The name Monera is best forgotten. Haeckel invented it for mythical organisms with contractile protoplasm and no nucleus that probably never existed. All Haeckel's candidates turned out to be amoeboid protozoa where the nucleus had escaped detection by available primitive microscopes or in one instance an artifactual chemical precipitate in sea water. Naming prokaryotes Monera, as Copeland did just because they lack a nucleus, is misleading as they lack contractile cytoplasm. I use the oldest name, Bacteria, known to most laymen, for the kingdom comprising all prokaryotes, following the first proponent of bacteria as a distinct kingdom (Enderlein 1925). Contrary to Haeckel's ideas, the most primitive surviving organisms are not contractile slime blobs, but rigid bacteria. Bacteria generally have rigid cell walls, never the branching filaments of actin protein that form an internal skeleton for all eukaryote cells. Bacteria equally lack the motor protein myosin that actively moves along actin filaments, causing the contraction of muscles, movements of amoebae and slime moulds and internal movements in all eukaryote cells including those of plants and fungi that evolved cell walls secondarily—entirely independently of each other and bacteria.

The origin of actin and myosin from known bacterial precursors was central to the origin of the eukaryote cell; as Stanier (1970) first suggested and I explained in detail (Cavalier-Smith 1975, 1987*a*, 2002*b*, 2009*b*, in press), numerous radical innovations in cell structure that made eukaryotes were tied up with the origin of predation on other cells by engulfing them by phagocytosis, an ancestral property for protozoa and animals. By contrast, no bacteria can eat other cells by engulfment, though several groups of bacteria became predators by evolving enzymes to digest prey externally, just as do some fungi and carnivorous plants.

Erasmus and Charles Darwin's fascination with insectivorous plants such as sundews and pitcher plants and with climbing plants probably stemmed from seeing them as potential missing links between the plant and animal kingdoms, offering clues how animals, classically characterized by eating and motility, might have evolved from plants. However, the evolutionary link between animals and plants is indirect, via unicellular protozoa. The carnivorous habits of certain plants and fungi arose entirely independently of those of animals, though the secretory mechanisms of their digestive enzymes evolved in their protozoan common ancestors—many of the enzymes themselves dating back still earlier to their bacterial ancestors. Although bacteria and protozoa were discovered long before, in 1675 (van Leeuwenhoek 1677),^1^ the central importance of unicellular organisms for reconstructing deep phylogeny was only obvious after the lingering notion of ongoing spontaneous generation (which Barry (1843) and Leeuwenhoek denied) was more decisively rejected in the 1860s by Pasteur. This reinforced the recognition of universal cell lineage continuity in 1852 by Remak and Virchow (Baker 1953). Virchow (1859) popularized the much earlier dictum ‘omnis cellula e cellula’ more influentially in the very year, 1858, when Darwin and Wallace (1858) publicized natural selection. The later elucidation of chromosome structure, mitosis and meiosis effectively proved the monophyly of the eukaryote cell, allowing Weismann (1889) to portray multicellular organisms as lineages of adhering cells within which vertical inheritance dominated and the germ line is relatively immune from environmental influences and direct effects of use and disuse, and paved the way for proper interpretation of Mendelian ratios.

**Figure 1.**
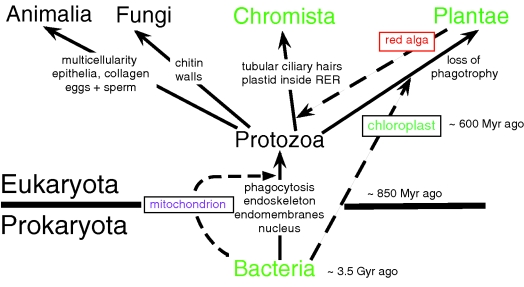
The six-kingdom, two-empire classification of life. Three major lineage mergers (symbiogeneses involving cell enslavement after phagocytic engulfment) are shown as dashed lines; four additional mergers that transferred chloroplasts from green plants or chromists into different protist lineages to make novel kinds of algae (Cavalier-Smith 2007*c*) are omitted for clarity (figure 6). The ancestrally photosynthetic kingdoms (Bacteria, Plantae and Chromista) are in green, but in each many lineages have lost photosynthesis. Chloroplasts originated when a biciliate protozoan internally enslaved a cyanobacterium bounded by two membranes to become the first plant. Chloroplasts are in the cytosol in Plantae, but inside two extra membranes in most Chromista: the ex-plasma membrane of the enslaved red alga, plus an RER membrane. Photosynthetic chromists include brown seaweeds, diatoms, haptophytes and cryptomonads. To portray early evolution in more detail, one must expand the two ancestral kingdoms by subdividing them more finely, as in figures 3 and 4 for Bacteria and figures 4 and 6 for Protozoa. But showing such basal groups in a phylogenetic tree as a single paraphyletic taxon, as here, is perfectly permissible and better focuses on the major steps in progressive evolution that generated the kingdoms than would excessive subdivision into a forest of ancient branches.

The virtual universality of the genetic code and pervasive sequence similarity of numerous genes and of central biochemical pathways in all organisms have proved the monophyly of all life. The unity of cell machinery for inserting nascent proteins directly into membranes and of key membrane proteins shows that all cells are lineal, vertical descendants of the very first cell (Cavalier-Smith 2001, 2006*c*), notwithstanding the dramatic structural differences between bacteria and eukaryotes or the unique membrane lipid chemistry of archaebacteria—which evolved from conventional bacteria (eubacteria) by lipid replacement, not independently from membrane-free naked-gene precursors as has sometimes been claimed. Figure 1 emphasizes that the fundamental differences between plants, animals and fungi reflect their independent origins from unicellular protozoan ancestors.

Lamarck (1809) insightfully contrasted the true natural order of life (what we now call phylogeny) with all classifications of life into discrete groups, which he correctly viewed as artificial human creations for our ends. Classification's purpose is not to ‘represent genealogy’ (that is the purpose of phylogeny) but to establish named coherent groups that are sensibly distinguishable from other groups, ideally by common ancestrally shared features, in an evolutionarily sound hierarchical system (Cavalier-Smith 1998). Taxonomy necessarily involves simplification and judgement about which characters to emphasize for human comprehension of biodiversity, without overtaxing our brains by its immensity. It cannot be done by algorithms delegatable to computers or inexperienced graduate students. Phylogenetically, even ‘biological species’ are artificial, as they are not unambiguously demarcated from their ancestors—except for allopolyploids, the only taxon that arises instantaneously.

## 5. Grades, clades and the big picture of organismal evolution

All six kingdoms are monophyletic in Haeckel's classical sense, i.e. each arose by one major evolutionary transformation (figure 1). For the origin of plants, the transformation was the enslavement by a protozoan of a phagocytosed cyanobacterium, turning it into a chloroplast by evolving novel membrane proteins able to extract chemicals for the host's benefit and evolving a novel protein-import machinery that targets such proteins to the chloroplast (Cavalier-Smith 1982, 2000). Thus, unbeknown to Haeckel, Darwin or Weismann, the tree of life involves not only divergence of cellular lineages, but on extremely rare occasions also mergers of distantly related lineages into one evolutionarily chimaeric cell. Such cell enslavement and profound integration, called symbiogenesis by Mereschkovsky (1910) who first proposed it for chloroplasts (Mereschkovsky 1905), yields more dramatic innovation than can mutation and selection alone. Symbiogenesis is analogous to Empedocles’ almost 2500-year-old idea of evolution by chimaera formation among body parts, impossible for multicells but not for unicells; of course, each symbiogenesis also involves thousands of mutations and their selection through benefiting host reproductive success. Symbiogenesis much more profoundly influenced megaevolution than did sex, which arose during the origin of eukaryotes, enabling closely related cells to fuse and pool their genetic and other resources—mainly of microevolutionary significance. Sex on the microscale and much rarer symbiogenesis reticulate cell lineages over history, making nonsense of cladistic dogma (Hennig 1966) assuming only divergence without mergers. LGT which occurs occasionally in Protozoa and plants independently of symbiogenesis (Keeling & Palmer 2008), but extremely seldom in animals because of their segregated germ line, and rather commonly in bacteria (Doolittle *et al*. 2003), also invalidates a purely cladistic vertical inheritance model for evolutionary history. Real evolution often ignores Germanic cladistic logic; its messiness and lack of rules makes classification an art where compromise is necessary and rigid formalism harmful.

Nonetheless, the distinction between a terminal branch of the evolutionary tree (a clade), e.g. animal or fungal kingdoms, and a basal, ancestral segment of the tree, e.g. Protozoa or Bacteria (each a distinctive grade of organization, not a clade), is important, especially when discussing extinction and origin of groups. Haeckel (1868) introduced cladus as a taxonomic category just below subphylum, but Huxley (1957, 1959) gave clade the general meaning of any monophyletic branch of the evolutionary tree. He did so when contrasting the two fundamental ‘vertical’ phylogenetic processes: cladistic splitting of lineages and progressive change along a lineage (among ‘horizontal’ phylogenetic processes, only sex was then appreciated, symbiogenesis and LGT being unproven). Huxley, like Darwin and Haeckel, correctly emphasized the equal importance of splitting and progressive change for understanding phylogeny and evolutionary history. Oddly, the school of ‘phylogenetic systematics’ founded by Hennig (1966) grossly downplayed the phylogenetic importance of progressive change compared with splitting, seen by them as so all-important that many Hennigian devotees dogmatically insist that ancestral groups like Bacteria, Protozoa and Reptilia be banned. Hennig called such basal groups with a monophyletic origin ‘paraphyletic’ and redefined monophyly to exclude them and embrace only clades, likewise redefined as including all descendants of their last common ancestor. This redefinition of ‘clade’ is universally accepted, but Hennig's extremely confusing and unwise redefinition of monophyly is not. Though accepted by many, sadly probably the majority (especially the most vociferous and over self-confident, and those fearful of bullying anonymous referees, of whom I have encountered dozens mistakenly insisting without reasoned arguments that paraphyletic taxa are never permissible), it is rightly firmly rejected by evolutionary systematists who consider the classical distinction between polyphyly and paraphyly much more important than distinguishing two forms of monophyly (paraphyly and holophyly, using the precise terminology of Ashlock (1971), where holophyletic equals monophyletic *sensu* Hennig).

Monophyly and polyphyly were invented to clarify origins; distinguishing between paraphyly and holophyly has nothing to do with the origin of a group, but with how taxonomists cut up the continuous phylogenetic tree into discrete named units. The phrase ‘paraphyletic origin’ that one sometimes sees is conceptual nonsense. This controversy is much more fundamental and broadly biologically important than a mere difference in preferred nomenclature. It reflects a pervasive difference in philosophy; excluding ancestral groups from the concept of monophyly perverts Haeckel's evolutionary definition. I agree with Mayr (1974), Halstead (1978) and others that the Hennigian perspective impedes realistic scientific discussion of phylogenetic history, because of its evolutionarily unrealistic formalism based on an intellectually impoverished view of the complexities of actual phylogenies, especially its failure to come to grips with evolutionary transformation, the reality of ancestors, and not least its dogmatism.

Let me illustrate the importance of distinguishing polyphyly and paraphyly by considering the case of Fungi and Pseudofungi in relation to figure 1. Classically, oomycetes, e.g. *Phytophthora infestans* that caused the 1844 Irish potato famine, were considered fungi, being included in kingdom Fungi in Whittaker's (1969) five-kingdom system. We are now certain that oomycetes are actually more closely related to heterokont algae (e.g. diatoms, brown seaweeds) and belong with them in the superphylum Heterokonta within the kingdom Chromista; they belong with hyphochytrids (also once wrongly considered fungi) and the phagotrophic flagellate *Developayella* in the heterokont phylum Pseudofungi (Cavalier-Smith & Chao 2006). Cellulose walls, often-filamentous body forms, and saprotrophic or parasitic lifestyles of oomycetes, which led to their incorrect classification as fungi, evolved entirely independently from the chitinous walls, hyphae and saprotrophy of true fungi. Oomycetes and fungi evolved independently from naked unicellular heterotrophic eukaryotes that fed by phagocytically engulfing prey. As similarities between fungi and pseudofungi are convergent and relatively superficial, a ‘fungoid’ group embracing both but not protozoa would be polyphyletic and unacceptable as a taxon. (Interestingly, a fair number of genes appear to have been laterally transferred from fungi to pseudofungi, which might have played a minor role in their convergence (Richards *et al.* 2006).)

**Figure 2.**
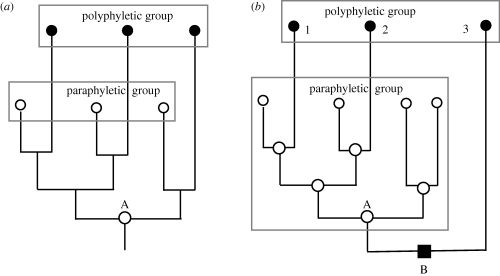
Contrasts between paraphyletic (ancestral) and polyphyletic groups. (*a*) The special case used by Hennig (1974) to claim that there is no cladistic difference between them because both have the same common ancestor (A) and an identical ancestral branching pattern. (*b*) A more realistic case where the three black-circle taxa do not have the same last common ancestor as the white-circle group, but have a different last common ancestor (B) which also has a different phenotype (black square) from A and from themselves. Case (*b*) shows that Hennig's claim for cladistic equivalence between paraphyletic and polyphyletic taxa lacks generality and rested on a cunningly chosen exceptional example. A paraphyletic group includes its last common ancestor and a polyphyletic one does not, a key fact partially concealed by Hennig misleadingly putting the same-sized box around both groups; to have correctly represented paraphyly the lower box should have included A, as it does in (*b*), where the obvious monophyly (single origin) of the paraphyletic white-circle taxon is much clearer than in Hennig's tendentious figure. The figure on the right also more strongly makes the point that the difference between polyphyly and paraphyly lies in the shared defining character (white circle) of the paraphyletic group having had a single origin, whereas the shared defining character of the polyphyletic group had three separate origins, i.e. a strongly contrasting phylogenetic history. Moreover, in (*b*) taxa 1 and 2 evolved black circleness in parallel from separate but phenotypically similar white-circle ancestors, whereas taxon 3 evolved it convergently from a cladistically and phenotypically more distinct black-square ancestor. It should be obvious that classifying white-circle taxa together is phylogenetically sound, i.e. they have a shared white-circle history, whereas classifying the black-circle ones together is unsound—being strongly contradicted by the lack of shared black-circle history. Unlike (*a*), (*b*) is a proper phylogeny with all ancestors and phenotypes shown; ignoring ancestral phenotypes makes nonsense of phylogeny. Cladistic aversion to paraphyletic groups, and lumping of paraphyly and polyphyly as ‘non-monophyly’, are logically flawed and anti-evolutionary (see also Cavalier-Smith (1998) which explains that clades, grades and taxa are all useful but non-equivalent kinds of group and that all taxa need not be clades and all clades need not be taxa).

In marked evolutionary contrast to the polyphyletic fungoids, the shared common features of an ancestral (paraphyletic) group like Protozoa evolved once only and were inherited continuously from a common ancestry, making their similarity much more fundamental and unified. The naked phagotrophic lifestyle and often flagellate and/or amoeboid motility of most members of kingdom Protozoa evolved once only in their last common ancestor, as part of an extremely complex set of over 60 major innovations, the most radical in the history of life (Cavalier-Smith 2009*b*). Thus, the evolutionary status of polyphyletic groups such as fungoids and paraphyletic ones such as protozoa differs radically; recognition of the important contrast between them (figure 2) depends on correctly deducing the phenotype of common ancestors. Even fungoids ultimately had a last common ancestor (but one of non-fungoid phenotype); it so happens that it was also the common ancestor of all four derived (holophyletic) eukaryotic kingdoms and the paraphyletic subkingdom Sarcomastigota of the paraphyletic kingdom Protozoa. An analogous purely hypothetical example of shared common ancestry led Hennig (1974) to assert that there is therefore ‘no difference’ between paraphyletic and polyphyletic groups ‘in the structure of their genealogical relationships’ (figure 2*a*). His implication that distinguishing paraphyly and polyphyly is therefore unimportant for systematics or arbitrary does not remotely follow; it merely underlines the casual neglect of actual ancestors and their phenotypes, and differing degrees of phenotypic change generally, by Hennigian cladistic philosophy. There being in these instances a shared common ancestor between a paraphyletic and a polyphyletic group does not nullify the importance of the distinction, which depends entirely on the historical phenotypes along the stems of the phylogenetic tree (figure 2), and not on the branching order. I am repeatedly irritated when indoctrinees of Hennig's narrow, biased viewpoint assert that they have shown a taxon to be ‘non-monophyletic’ (they mean non-holophyletic); this umbrella term conflates paraphyly and polyphyly—evolutionarily very different and of contrasting taxonomic implications. Polyphyletic taxa must be split into monophyletic ones; a paraphyletic one already is monophyletic in phylogenetic origin and need not necessarily be abandoned or radically revised, though sometimes this is advisable if it is excessively heterogeneous. Non-monophyletic conceals information, contrary to Hennig's wish to make terminology more informative and precise.

Hennig (1974) insisted that a monophyletic group must not share a last common ancestor with another monophyletic group. He would reject calling protozoa monophyletic because their last common ancestor is identical to that of Eukaryota—a silly argument because protozoa has lower rank within the more inclusive Eukaryota. Comparing equally ranked taxa, each of the five eukaryotic kingdoms has a different last common ancestor. Evolutionary classification, which I and many other taxonomists practice, recognizes the reality of ancestors and the importance in principle of classifying them; thus, the last common ancestor of a monophyletic group is always included in the taxon, so every taxon corresponds with a single segment of the evolutionary tree having only one species at its base (figure 2*b*). One cannot emphasize too strongly that a Hennigian cladogram is not a phylogenetic tree; cladograms have no ancestors, only extant species. Sequence trees are also not phylogenetic trees of organisms, being agnostic about ancestral phenotypes. Hennig (1974) and many followers condemned using degree of phenotypic difference in phylogeny and classification because there is no single objective measure of it. This stupidly throws the taxonomic baby out with the bathwater. The very reason we wish to classify organisms is their phenotypic differences, not their genealogical history. If all organisms had the same phenotype but a known genealogical history, it would be pointless to classify them by subdividing the tree into named pieces. Cladistic reasoning uses groups defined by phenotypic differences, so is just as sensitive as evolutionary taxonomy to there being no quantitative scale for them.

Do not misinterpret me as claiming that the distinction between the two types of monophyly (paraphyly and holophyly) is unimportant. For some purposes it may be, but in two situations the distinction is crucial: when discussing extinction or origins.

It is well known that discussions of group extinction must distinguish between real extinction of a holophyletic group such as trilobites, which genuinely left no descendants, and pseudoextinction of a paraphyletic group such as dinosaurs, which left descendants (birds) that differ so greatly from the ancestral group that they are not classified within it.

**Figure 3.**
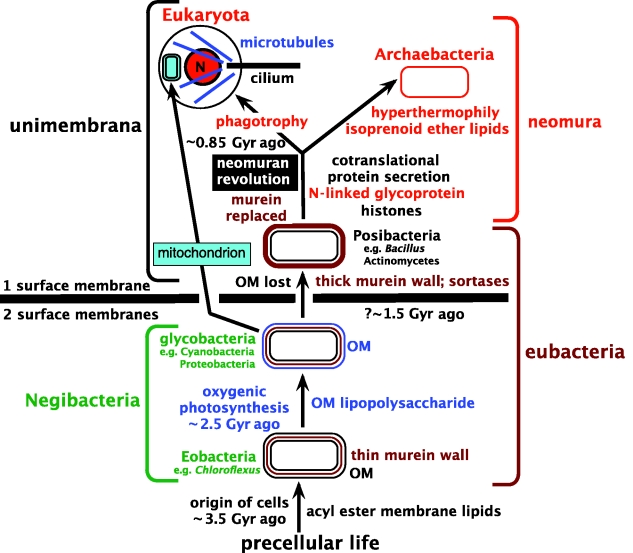
The tree of life, emphasizing major evolutionary changes in membrane topology and chemistry. The most basic distinction is between ancestral Negibacteria, with a cell envelope of two distinct lipid bilayer membranes, and derived unimembrana, with but one surface membrane. Negibacteria were ancestrally photosynthetic (green), while unimembrana were ancestrally heterotrophs. A photosystem duplication enabled oxygenic photosynthesis (approx. 2.5 Gy ago: Kopp *et al*. 2005; Kirschvink & Kopp 2008) roughly when the outer membrane (OM) dating from the first cell acquired novel impermeable lipopolysaccharide and transport machinery. The late date of the neomuran revolution involving 20 major novelties is based on morphological fossils of eukaryotes and the argument that archaebacteria cannot be substantially older than their eukaryote sisters (Cavalier-Smith 2006*a*,*c*). Eubacteria, characterized ancestrally by cell wall murein, is an ancestral paraphyletic group that I do not make a taxon because I rather subdivide bacteria into subkingdoms Negibacteria and Unibacteria (comprising the phyla Posibacteria and Archaebacteria; figure 6), as their differences in membrane topology are more fundamental and significant (and more rarely change) than wall chemistry. Neomura is an important named clade that I chose not to make a taxon to avoid conflict with the much more radical differences between bacteria and eukaryotes. This exemplifies the principle that taxonomists should (and generally do) choose points on the continuous phylogenetic tree of maximal phenotypic disparity for artificially cutting it into taxa—NOT points of greatest cladistic depth irrespective of phenotype. Taxa have an initial capital; grades and clades that are not taxa have lower-case initials. Previously, hydrocarbon biomarkers were misinterpreted to give much earlier dates for eukaryotes and cyanobacteria, but these are invalidated by isotopic proof of hydrocarbon mobility from much younger strata (Rasmussen *et al*. 2008). Justification for the topology of this tree and its being correctly rooted and thus historically correct is elsewhere (Cavalier-Smith 2006*a*,*c*; Valas & Bourne 2009). A widespread contrary view that the root is between eubacteria and neomura stems from protein paralogue trees with long-branch topological artifacts and ignoring palaeontological evidence that negibacteria are immensely older than eukaryotes. For simplicity, the fact that the nucleus (N) has a double envelope that is part of a pervasive endomembrane system is not shown. The ancestral eukaryote is shown with a single cilium and centriole, but both had probably doubled in number prior to the earliest divergence among extant eukaryotes (Cavalier-Smith submitted *b*).

It is less widely appreciated that when considering origins or reconstructing ancestral characters treating a paraphyletic group as holophyletic causes even more serious misinterpretations. Most papers discussing the nature of the ancestral cell make this very mistake by treating eubacteria as holophyletic, whereas they are almost certainly paraphyletic (see §§7–9). As figure 3 indicates, eubacteria are much more structurally diverse than archaebacteria. Contrary to widespread practice, I do not treat them as a taxon—not because it would be paraphyletic, but because the contrast between cells with two bounding membranes (Negibacteria) is more fundamentally important than the differences between Posibacteria and Archaebacteria, derived phyla which I have grouped as subkingdom Unibacteria (Cavalier-Smith 1998). Lumping all three eubacterial groups of figure 3 as one taxon conceals the profound importance of their structural differences; wrongly treating it as holophyletic makes it impossible to reconstruct the last common ancestor of life correctly. Most biologists since Iwabe *et al*. (1989) and Gogarten *et al*. (1989) have assumed that the root of the tree lies between neomura and eubacteria, but there is no sound evidence for this; more protein paralogue trees place the root within eubacteria, as in figure 3, than between eubacteria and neomura (making paralogue rooting self-contradictory and unreliable despite its attractions in theory: Cavalier-Smith 2006*c*). Arguments based on cell evolution and the fossil record strongly indicate that eubacteria are ancestral to neomura and thus paraphyletic (Cavalier-Smith 2006*a*,*b*). By failing to recognize this, most who discuss the last common ancestor of all life have reached entirely incorrect conclusions about its nature and do not even realize the necessity of deciding where the root of the tree really is within the immensely diverse eubacteria before deducing the ancestral phenotype. This error vitiates the conclusions of hundreds of papers. It also misled a generation of researchers into thinking that studying archaebacteria is especially relevant to the origin of life, which is not so if eubacteria really are paraphyletic.

To avoid such misinterpretation of paraphyletic groups, we need not abolish them; it is sufficient to flag them as paraphyletic (if we know that) and to teach biologists to use phylogenies directly, not classifications, for evolutionary reasoning about origins and extinction.

## 6. Evolutionists must be allowed to classify, rank and discuss ancestral groups

The amount of modification which the different groups have undergone has to be expressed by ranking them under different so-called genera, subfamilies, families, sections, orders and classes.

(Darwin 1859, p. 352)

The evolutionary unreality of Hennig's antipathy to ancestral taxa is highlighted by allopolyploidy, by the nature of bacteria and by symbiogenesis.

Allopolyploidy involves lineage fusion that is beyond the scope of cladistics, which unrealistically assumes only divergence. The view of Linnaeus and Erasmus Darwin (1794, p. 507) that most species arose by hybridization is wrong. Yet allopolyploidy is common, especially in flowering plants; two historically distinct species (sometimes from different genera) hybridize and the typically sterile hybrid becomes fertile by ploidy doubling. The resulting allopolyploid is an instantaneously evolved new species of novel phenotype, unable to breed with either parent. Several cases have been observed in nature (e.g. *Spartina anglica*, *Primula kewensis*; Haldane 1932; Baumel *et al*. 2001; Salmon *et al*. 2005), proving that species sometimes evolve just as Linnaeus and Lamarck thought. Both parent species generally survive and are unchanged by the origin of the third new species, which invalidates the biologically nonsensical cladistic dogma that a sister group to a new species must be considered a different species from its parent even if phenotypically identical. Moreover, both ancestral species are valid taxa, despite being paraphyletic; typically, each has at least some derived characters not shared by their descendant holophyletic allopolyploid species. Thus another spurious justification of antipathy to paraphyletic taxa is mistaken, i.e. that they have no characters not also shared by their derivatives.

The falsity of this dogma is still more strikingly shown by the kingdom Bacteria, which has several universal positive characters never found in eukaryotes, their descendants. This came about because the drastic nature of eukaryogenesis destroyed many bacterial synapomorphies that arose in the last common ancestral bacterium (Cavalier-Smith 1981, 1991*a*, 2009*b*, in press). This is often not so; many paraphyletic groups lack obvious ancestral characters not shared with any descendant group. Thus, when fungi and oomycetes evolved, ancestral protozoan phagotrophy was lost through the origins of their cell walls, but when chromists evolved phagocytosis was not lost, the cells remaining naked. Instead, a key event in the origin of Chromista was the fusion of the phagocytic vacuole membrane containing the enslaved red alga with the nuclear envelope, placing it and its plastid ever afterwards inside the rough endoplasmic reticulum (RER), giving plastid-bearing chromists a cell membrane topology fundamentally different from both of their ancestral groups: Plantae and Protozoa (Cavalier-Smith 1986, 2003, 2007*c*). The ancestral chromist was a mixotroph; it photosynthesized and phagocytosed prey, as several groups of naked chromistan algae still do (e.g. many chrysophytes, haptophytes and pedinellids) placing them outside the familiar animal–plant dichotomy. But other chromistan algae evolved cell walls (e.g. brown algal cellulose walls and diatom silica shells), dispensing with phagotrophy and becoming purely phototrophs. Other chromists abandoned photosynthesis, relying either on their ancestral phagotrophy (becoming phenotypically confusable with protozoa: zooflagellates, e.g. the heterokonts *Developayella*, *Cafeteria* and many Cercozoa; the giant pseudopodial ‘heliozoan’ *Actinosphaerium*; the whole infrakingdom Rhizaria ancestrally characterized by net-like pseudopodia and the centrohelid heliozoa; see Cavalier-Smith submitted *b*) or on saprotrophy like oomycetes or *Blastocystis*, a walled anaerobic human gut parasite (confusable with fungi; Cavalier-Smith & Chao 2006). Thus, chromist evolution was messy because of multiple independent losses of ancestral characters. For generations, this concealed their unity and distinctiveness from Protozoa and Plantae.

Naming Hennigian formalism ‘phylogenetic systematics’ was extremely misleading, as it focuses on only one of the two vertical processes of evolution (splitting), ignoring and contradicted by all three modes of horizontal evolution that make the true universal phylogeny a reticulating net, not an ever diverging tree (sex/allopolyploidy, symbiogenesis and LGT); if we remember that branches of real trees sometimes fuse, tree metaphors are useful.

I agree with cladists’ criticisms that Simpson's (1961) redefinition of classical monophyly was bad. His criterion that descent of a group from ‘one immediately ancestral taxon of the same or lower rank’ suffices for monophyly is far too loose. Such woolliness would allow animals and plants to be classified together in a supposedly ‘monophyletic’ kingdom merely because both evolved from Protozoa, despite their evident independent origin from two entirely separate groups of protozoa, or grouping birds and mammals because they evolved from reptiles. Though neither Simpson nor any sensible taxonomist would do either, the defence of Simpson by Mayr & Ashlock (1991) was illogical and counterproductive. Much more precision is needed, attainable by insisting that monophyly requires descent from a single ancestral *species* itself classified within the group in question as its first species (Mayr & Ashlock 1991), as classical taxonomists did long before Hennig (Mayr 1942). Simpson was probably led into that woolliness by problems in applying synapomorphies for extant mammals (hair, lactation and penis) to fossils and substituting a surrogate definition based on ear ossicle evolution that exhibits parallelism within reptiles. With respect to reptiles, Hennig (1974) accepted that many would regard his antipathy to Reptilia being a taxon as ‘shocking and absurd’; he even wrote with respect to the great magnitude of the differences between birds and mammals and their ancestral reptiles, the reason for treating each as distinct classes, that ‘it seems pure formalism, and perfectionism transcending any reasonable purpose to neglect these facts in a hierarchical system’. Well said. I would change ‘seems’ to ‘is’.

Recognizing paraphyletic groups like bacteria and protozoa facilitates evolutionary discussion of how major groups arose from an ancestral group and of major advances in evolution such as the origin of eukaryotes from bacterial ancestors. If we are not allowed to classify and name ancestral groups, rational discussion of such evolutionary advances is severely hampered. Cladism deals only with sister relations, but evolution and phylogeny require analysis of ancestor descendent relationships, which is greatly impeded by the straitjacket of an exclusively cladistic perspective. Its linguistic and conceptual harmfulness is illustrated by the recent fashion among cladists to reclassify tetrapods, including themselves, as Osteichthyes—bony fish. It would be impossible to express the truth that tetrapods evolved from a bony fish if we call tetrapods bony fish; moreover, to call birds or elephants bony fish is stupid; such is the reductio ad absurdum of Hennigian nomenclatural dicta by some cladists. A cladist actually asserted at a meeting that ‘trees and humans are flagellates’—just because both ultimately descended from flagellate protozoa. Such quirky attitudes make discussion of real phylogeny impossible. If the name of an ancestral group should expand to embrace all descendant clades, the logical conclusion would be to make all organisms bacteria, all eukaryotes protozoa and (should sponges be confirmed as paraphyletic) all animals sponges; logically consistent cladists of that sort must accept that they are bacteria. If not, they should accept that such nomenclatural dogmas are deeply flawed, harmful to biology and abandon them forthwith. A philosophy that evades the reality of ancestors and denies the validity of ancestral groups is wrongly called ‘phylogenetic systematics’.

The reality of stasis and rarity of major transitions make it imperative to name ancestral groups for sensibly discussing progressive evolution. Showing decisively that pennate diatoms evolved from centric diatoms by changing cell symmetry and evolving a sternum was an important evolutionary advance (Mann & Evans 2007), not a taxonomic problem as myopic cladists misrepresent it (Williams 2007). There is no need whatever to abandon centric diatoms as a taxon because it is paraphyletic; it had a single origin and has an unambiguously definable phenotype not shared by any pennates. The idea that evolutionists and taxonomists must express relationships only in terms of sister groups, never parent or descendant groups, is most harmful. Citing Darwin to support a thesis does not prove it right (like everyone he made mistakes), but he undeniably accepted ancestral groups. His famous *Origin* diagram was a proper phylogenetic tree with labelled ancestors, some explicitly called parent genera, not a cladogram. He would surely consider banning paraphyletic taxa an absurd impediment to evolutionary discussion and comprehensive classifications that include fossils and ancestors (Cavalier-Smith 1998).

## 7. The four major kinds of bacteria

For the layman and lower schoolchild, six kingdoms are sufficient to summarize the diversity and history of life in a readily graspable manner (figure 1). For a biologist interested in deep phylogeny, each kingdom must be subdivided. Consider first Bacteria, where even a non-microbiologist wishing to understand early evolution of life only in relatively broad terms ought to appreciate the fundamental differences between four major types of cell (figure 3). Bacteria comprise two subkingdoms of contrasting membrane topology: Unibacteria and Negibacteria (figure 3).

Unibacteria have one surface membrane, like eukaryote cells that evolved from them; they comprise two phyla with radically different membrane chemistry: the ancestral Posibacteria and the derived Archaebacteria. Posibacteria have essentially the same membrane chemistry as eukaryotes: phospholipids with a glycerol backbone and two fatty acids attached by ester links (i.e. acyl ester lipids). Archaebacteria are sisters to eukaryotes (Cavalier-Smith 1987*a*, 2002*a*, 2006*c*; Yutin *et al*. 2008), not as often incorrectly thought their ancestors (Van Valen & Maiorana 1980; a recent paper favouring an ancestral rather than a sister relationship (Cox *et al*. 2008) is unconvincing, as within eukaryotes the topology of all their trees is wrong in half a dozen ‘statistically well-supported’ respects, indicating that the data and methods fail to reconstruct trees accurately for these branches, making it unwise to accept that the reported sister relationship between eukaryotes and crenarchaeotes is less suspect than these known errors). Unlike eubacteria and eukaryotes, archaebacteria make their membranes of glycerol phospholipids in which isoprenoids are attached by ether links to the glycerol backbone, which also has a novel stereochemistry. These novel lipids arguably arose to enable hyperthermophily—tolerating extremely high temperatures, sometimes over 100°C, when the ancestral archaebacterium replaced acyl esters by stabler isoprenoid ethers (Cavalier-Smith 1987*a*,*b*, 2002*a*, 2006*c*). This replacement enabled covalent bonds to link phospholipids in the two leaflets of the cytoplasmic membrane into a single layer, enabling these specialized bacteria to colonize the highest temperature habitats available on Earth in geothermally active areas. This specialization arguably also involved loss of many ancestral enzymes unable to cope with such extremes and to a greatly reduced genome size of archaebacteria compared with their posibacterial ancestors and eukaryotic sisters (Cavalier-Smith 2002*b*, 2007*a*). Later, some archaebacterial lineages, notably halobacteria, colonized similarly previously unexploited but lower temperature habitats, reacquiring many enzymes suitable for mesophilic habitats by LGT from eubacteria and making their membranes more fluid by losing covalent bonding between the bilayers (Cavalier-Smith 2002*b*). No unibacteria are photosynthetic fixers of carbon dioxide. None contain chlorophyll, though some halobacteria use sunlight trapped by carotenoids related to visual purple of animal retinas to make ATP.

Negibacteria, in marked contrast, are bounded by two membranes and are often photosynthetic, five different phyla containing members able to fix carbon dioxide using energy trapped by chlorophyll (Cyanobacteria; typically generating oxygen) or bacteriochlorophyll (Chlorobacteria, Sphingobacteria, Proteobacteria, Eurybacteria; all anoxygenic—never generating oxygen). Their inner bounding membrane is homologous with and ancestral to the bounding membrane of unibacteria and eukaryotes, having typical acyl ester phospholipids (except in Chlorobacteria) as in eukaryotes and Posibacteria and related membrane proteins. Their outer membrane (OM) has more variable chemistry, the basis for classification into two infrakingdoms: Eobacteria and Glycobacteria (Cavalier-Smith 2006*c*). The glycobacterial OM is homologous with and ancestral to the OM of mitochondria and chloroplasts but was lost in the ancestral unibacterium (Cavalier-Smith 2006*c*). Membrane continuity throughout evolution since before the origin of the last cell is a very basic evolutionary principle (Cavalier-Smith 1987*a*,*b*, 2001, 2004); as Blobel (1980) put it, ‘omnis membrana e membrana’. Membrane multiplication involves membrane heredity (Cavalier-Smith 2001, 2004) in which the different genetic membranes of a single cell are perpetuated by lipid- and protein-insertion mechanisms and machinery of high specificity. Thereby the inner membrane and OM of negibacteria, mitochondria (Cavalier-Smith 2006*b*) and chloroplasts, and the plasma membrane, peroxisomes and endomembrane systems of eukaryotes, remain distinct over hundreds of millions of years, perpetuated by growth and division of membranes always of the same kind. At the molecular level, membrane heredity involves self-complementarity between targeted proteins and membrane-embedded receptor proteins (Cavalier-Smith 2000), just as DNA heredity depends on DNA self-complementarity and three-dimensional complementarity between DNA and DNA-handling enzymes, e.g. DNA polymerases.

**Figure 4.**
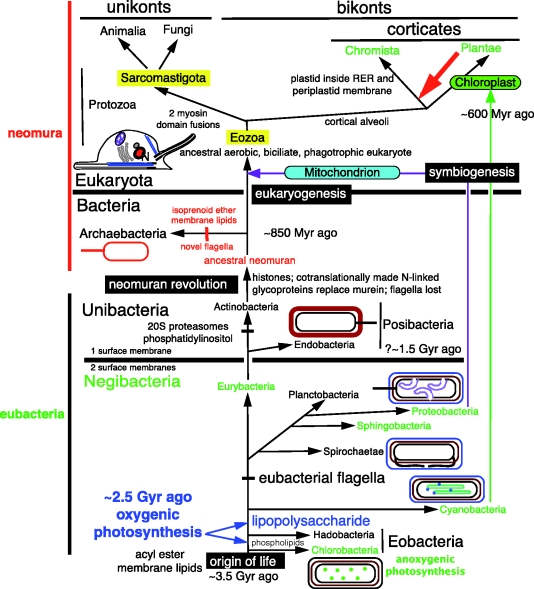
The tree of life, emphasizing the deepest branches. Ancestral groups of figures 1 and 3 are subdivided. Protozoa are resolved into two subkingdoms highlighted in yellow: the basal Eozoa (i.e. Euglenozoa plus Excavata), ancestrally characterized by a rigid cell pellicle supported by microtubules and the absence of pseudopodia (Cavalier-Smith submitted *b*) and the derived Sarcomastigota, ancestrally amoeboflagellates—probably with pointed pseudopodia, which gave rise to animals and fungi. Posibacteria comprise two subphyla: Endobacteria (putatively holophyletic) and Actinobacteria, which are probably the ancestors of neomura, having phosphatidylinositol lipids and proteasomes that both played key roles in eukaryogenesis (Cavalier-Smith 2009*b*). Glycobacteria are split into six phyla: three holophyletic, three paraphyletic (Cyanobacteria being ancestors of chloroplasts and thus partially of all Plantae, Chromista and those euglenoid eozoan Protozoa that secondarily acquired a plastid from green plants (figure 6); Proteobacteria being ancestors of mitochondria and thus in part of all eukaryotes; ancestral to Posibacteria are Eurybacteria). Eurybacteria include Thermotogales, Aquificales (now; see Bousseau *et al*. 2008), Heliobacteria and endospore-forming heterotrophs; they are often unwisely lumped with Endobacteria as ‘Firmicutes’ merely because they group on sequence trees, despite being structurally negibacteria. Ancestrally photosynthetic groups are in green. The ancestral (paraphyletic) Eobacteria are split into two putatively holophyletic phyla: Chlorobacteria (often photosynthetic, i.e. non-sulphur green filamentous bacteria like *Chloroflexus*) and the heterotrophic Hadobacteria (e.g. *Thermus*, *Deinococcus*). Bacteria ancestrally lacked flagella; soon after eubacterial rotary flagella evolved, one lineage relocated them to the periplasmic space to become spirochaetes (thumbnail sketch). Many lineages lost flagella, e.g. most Sphingobacteria and ancestors of neomura: archaebacteria re-evolved flagella and eukaryotes cilia, both entirely unrelated to eubacterial flagella. The higher proportion of holophyletic groups in figure 4 than figure 1 or 3 is bought at the expense of losing simplicity that more strikingly portrays major body-plan differences within eukaryotes (figure 1) and prokaryotes (figure 3). The extra cladistic resolution at the base of figure 4 is important for some purposes but irrelevant to others. Figures 1, 3, 4 and 5 are different ways of acceptably summarizing distinct aspects of the single true historical tree (which is reticulated and has ancestors and is thus not a cladogram or sequence tree). For more details on the 10 bacterial phyla and their relationships see Cavalier-Smith (2002*a*, 2006*c*). Oxygenic photosynthesis can have evolved no later than where shown by the upper blue arrow, immediately before the divergence of Cyanobacteria, but one reasonable non-decisive argument favours a marginally earlier origin before Hadobacteria diverged (lower blue arrow, when phospholipids arose; Cavalier-Smith 2006*c*). A sound hierarchical classification with ranks can simply represent both the fundamental shared similarities within ancestral groups, such as Posibacteria, Eobacteria, Bacteria and Protozoa, and the profound differences between their major subgroups.

In glycobacteria, the inner leaflet of the OM lipid bilayer comprises typical acyl ester phospholipids but the outer leaflet is made of much more complex and substantially more impermeable lipolysaccharides. Glycobacteria can therefore only grow because the OM also has cylindrical pores made of oligomeric *β*-barrel proteins called porins that allow nutrient uptake from the environment. Their OMs uniquely have other macromolecular complexes mediating exchanges of larger molecules across it. All photosynthetic bacteria except Chlorobacteria are glycobacteria. In Eobacteria (comprising Chlorobacteria and Hadobacteria), the OM is simpler, with lipolysaccharide absent; Eobacteria have glycolipids based on long-chain diols instead of glycerolipids (Pond *et al*. 1986; Woese 1987; Wait *et al*. 1997), unlike all other organisms. This chemical simplicity of eobacterial OMs is considered primitive, not a derived trait, in contrast to the topological simplicity of unibacterial membranes which arose by secondary loss of the OM as explained in detail elsewhere (Cavalier-Smith 2006*c*). Recent contrary claims that Posibacteria preceded Negibacteria are refuted by Valas & Bourne (2009). Porins and the universal mechanism for inserting *β*-barrel proteins into the OM apparently evolved prior to glycobacteria, as they are also present in Hadobacteria (heterotrophs such as the heat-loving *Thermus* and the extremely radiation resistant *Deinococcus* and their relatives). Ancestors of Hadobacteria, and of the purely heterotrophic glycobacterial phyla Spirochaetae and Planctobacteria, must have lost photosynthesis. Photosynthesis was also lost on several occasions within the holophyletic Chlorobacteria, Proteobacteria, Sphingobacteria and the paraphyletic Eurybacteria, arguably the ancestors of the non-photosynthetic Posibacteria (figure 4, which summarizes inferred relationships among the bacterial phyla forming the deepest branches in the tree of life). Only Cyanobacteria never lost photosynthesis and remain almost as uniform today in basic physiology as when they first evolved just over 2.4 billion years ago (though some fix nitrogen and some do not, several lineages lost their ancestral red/blue phycobilin pigments that makes them blue-green or red and one even lost photosystem II; Zehr *et al*. 2008).

Just a few major innovations and many losses created the patchwork of bacterial phenotypes seen across the tree; in addition, LGT complicated the story by introducing numerous archaebacterial thermophilic genes into eubacterial ancestors of the eurybacteria *Thermotoga* and *Aquifex* (Nesbø *et al*. 2001; Nesbø & Doolittle 2003; Bousseau *et al*. 2008), possibly endowing them with hyperthermophilic phenotypes that eubacteria might not have evolved without such foreign help. Eubacteria probably originated as the first cells in cool habitats where organic molecules would be most stable during the origin and early evolution of life (Cavalier-Smith 2001). Other useful enzymes have been transferred piecemeal among very distantly related bacterial lineages and from bacteria to eukaryotes (occasionally the reverse), but LGT never transferred major organismal properties dependent on numerous tightly interacting gene products, e.g. oxygenic photosynthesis, cell envelope structure or cell wall chemistry, from one bacterial lineage to another (only symbiogenesis by cell mergers managed that in eukaryotes). Non-laterally transferred morphology or macromolecular assemblies, being also immune to sequence tree reconstruction biases, were crucial for reconstructing the phylogeny of figures 3 and 4.

When mitochondria and chloroplasts evolved from enslaved cyanobacteria and *α*-proteobacteria (both glycobacteria), the OM lipolysaccharide was lost, being replaced by host phosphatidylcholine, but porins remained, some even being recruited for the novel protein-import machinery that made these enslaved bacteria integrated organelles. Porins are *β*-barrel proteins, a class of proteins absent from all membranes except the negibacterial OM and these two organelle OMs. Chlorobacteria are the only negibacteria that lack porins or other *β*-barrel OM proteins; they are therefore considered the most primitive form of cell and most ancient ancestral type of negibacteria, because one can rule out the alternative idea of secondary simplification by loss of *β*-barrel OM proteins because loss of ability to insert them is lethal (Cavalier-Smith 2006*c*); only if the OM was lost at the same time as when Posibacteria originated could cells already dependent on *β*-barrel proteins survive. Some evolutionary innovations are effectively irreversible, e.g. many during the origin of eukaryotes.

Much writing on bacteria lumps Negibacteria and Posibacteria together as eubacteria (sometimes the prefix eu- is dropped (Woese *et al*. 1990); extremely unwise and confusing). Eubacteria ancestrally had walls of the peptidoglycan murein, never present in archaebacteria (now sadly often called archaea to conceal their truly bacterial nature (Woese *et al*. 1990); established taxonomic names should not be changed merely to promote a partisan view). In eukaryotes, murein is present only in the envelope of glaucophyte chloroplasts, as a relic of the cyanobacterial ancestor of plastids lost in the common ancestor of green and red algae, thus absent from other eukaryotes (its persistence in glaucophytes alone for 600 Myr puts the lie to the inevitability of evolutionary change and to using such archaisms to argue for the recency of events). A major event in the history of life, second in its revolutionary importance only to the origin of the eukaryote cell, was the replacement of murein walls (which are covalently three-dimensionally cross-linked to form an encasing sacculus molecule bigger than the cell), by walls of N-linked glycoproteins, which are not thus interlinked. This replacement occurred in the common ancestor of eukaryotes and archaebacteria, which are therefore grouped as clade neomura (‘new walls’; Cavalier-Smith 1987*a*). Glycoproteins are potentially freely mobile in the fluid phospholipid surface membranes, which the ancestor of eukaryotes exploited by converting the wall into a flexible surface coat. Its sister ancestor of archaebacteria prevented mobility by evolving rigid isoprenoid ether lipids and a crystalline glycoprotein wall. Potential flexibility of the neomuran cell surface was a prerequisite for the origin of phagocytosis by prey engulfment (which indirectly made the eukaryote cell; Cavalier-Smith 2002*b*, 2009*b*) and sexual cell fusion.

Negibacteria are another ancestral group that universally shares a positive character (OM) absent in the clade (unimembrana) derived from it, which shares only the absence of the OM. As for Bacteria, this refutes cladistic dogma that paraphyletic groups are inadmissible because they necessarily lack positive traits that unify them.

## 8. The three-domain view of life is flawed: sequence trees are often misrooted

Many who use only RNA and protein sequences to interpret organismal history overlook the importance of the unique dramatic evolutionary transitions in cell structure in figures 3 and 4 for unravelling deep phylogeny. As I have shown (Cavalier-Smith 2002*a*, 2006*a*,*c*), ignoring organismal structure, cell biology and palaeontology led to a now widespread fundamental misinterpretation of the history of life, the three-domain system, in which it is incorrectly assumed that eubacteria are holophyletic and not substantially older than archaebacteria and eukaryotes and that the tree is rooted between neomura and eubacteria (Woese *et al*. 1990). These serious errors stemmed not only from failing to integrate sequence evidence with other data, but also from unawareness of the often extremely non-clock-like nature of sequence evolution and of grossly misleading systematic errors in sequence trees for molecules that do not evolve according to naive statistical preconceptions (see Cavalier-Smith 2002*a*, 2006*a*,*c*). Transition analysis using complex three-dimensional characters less prone to phylogenetic artefact than sequences provides powerful evidence that Posibacteria are ancestral to neomura, negibacteria ancestral to unibacteria and eobacteria ancestral to glycobacteria (Cavalier-Smith 2002*a*, 2006*a*,*c*; Valas & Bourne 2009). Palaeontology provides equally strong evidence that Cyanobacteria are substantially older than eukaryotes and that eubacteria are an ancient ancestral group, not a clade (Cavalier-Smith 2006*a*). Statistics cannot adequately model the historically unique exceedingly rare events of megaevolution, for which assumptions of uniformism are entirely invalid (Cavalier-Smith 2006*b*). However, taking into account their known and inferred biases, sequence trees are compatible with the position of the root, the directions of the transitions and the topology of figures 3 and 4.

## 9. The four ages of life

**Figure 5.**
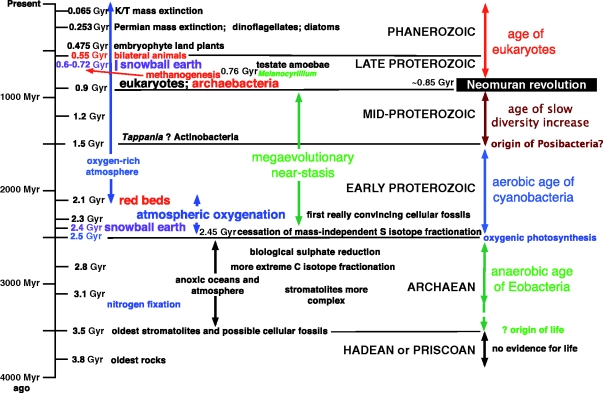
The four ages of life. The six geological eras (black capitals) are demarcated especially by their fossils, which are absent in the Hadean, extremely sparse and problematic in the Archaean, numerous after about 2.2 Gy but all microscopic in the Proterozoic, and of every size and abundant in the Phanerozoic. In recognizing four ages of life (lower case colour on the right), I group the Late Proterozoic and Phanerozoic eras as the age of eukaryotes, because the origin of eukaryotic and archaebacterial cells that immediately followed the neomuran revolution is much more fundamental than the origin of bilaterian animals (around 550 Myr ago; Martin *et al*. 2000) that arguably initiated the Cambrian explosion (approx. 535–525 Myr ago) at the base of the Phanerozoic. On this view, increased acritarch fossil complexity at the transition from mid- to late Proterozoic was directly caused by the origin of the eukaryote cell. The Archaean/Proterozoic boundary essentially corresponds with the origin of photosystem II and oxygenic photosynthesis, shortly before the divergence of cyanobacteria (which are holophyletic, ignoring their being chloroplast ancestors, and thus not directly ancestral to other photosynthetic glycobacteria; figure 4). The early to mid-Proterozoic boundary is the most difficult to connect to a specific biological innovation. It may correspond with the origin of the posibacterial cell by a massive thickening of the murein wall and consequent loss of the OM, which may have stimulated the colonization of primitive cyanobacteria-dominated soils by Posibacteria (Cavalier-Smith 2006*a*); identification of the most complex mid-Proterozoic fossils as fungi (Butterfield 2005) is not compelling (earlier suggestions of eukaryotic algae were even less convincing). Possibly they are pseudosporangia and hyphae of Actinobacteria (Cavalier-Smith 2006*a*). The first convincing eukaryotic fossils are *Melanocyrillium* testate amoebae (Porter & Knoll 2000), though I do not accept their overconfident assignment to extant protozoan phyla (Porter *et al*. 2003); more likely they are an extinct group of early eukaryotes (Cavalier-Smith 2009*a*). Except for the final Vendian Period, bearing arguably stem animal fossils not confidently assignable to extant phyla, the Neoproterozoic was an era of only protists (unicellular eukaryotes; prior to the origin of plastids, perhaps little over 600 Myr ago, probably mainly Eozoa (figure 6) and Amoebozoa) and bacteria; phagotrophs diversified and underwent symbiogenesis to make various eukaryotic algae.

The Phanerozoic is the age of macroscopic life, the Proterozoic the age of visible microscopic life and the Archaean eon the age of indirectly inferred life. Mapping the known cellular diversity and now reasonably well-established overall phylogeny of life (figures 3 and 4) onto the fossil record enables us to divide the history of life into four successive stages (figure 5).

**Figure 6.**
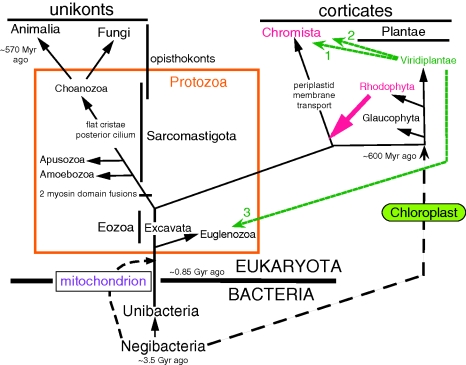
The eukaryote evolutionary tree, showing the messiness of real phylogeny. Compared with figure 1, the ancestral kingdoms Protozoa (all taxa inside the orange box) and Plantae are expanded to show their deepest branches and the reticulation caused by symbiogenetic cell enslavement. Apusozoa are gliding zooflagellates (Apusomonadida, Planomonadida; Cavalier-Smith *et al*. 2008) deeply divergent from other main groups. The large red arrow indicates the enslavement of a phagocytosed red alga over 530 Myr ago by a biciliate protozoan to form the chimaeric common ancestor of kingdom Chromista. Previously, Alveolata (i.e. Ciliophora and Myzozoa) were treated as protozoa, but are now included within Chromista (Cavalier-Smith submitted *b*); Ciliophora and most Myzozoa (subphyla Dinozoa, Apicomplexa) have lost photosynthesis (though many heterotrophic Myzozoa retain colourless plastids for lipid synthesis). Likewise, Rhizaria (Cercozoa, Foraminifera, Radiozoa) and centrohelid Heliozoa, both formerly treated as Protozoa, appear to be major chromist lineages that independently lost the ancestral red algal chloroplast and are now placed within Chromista not Protozoa (Cavalier-Smith submitted *b*). One small lineage of dinoflagellates (Dinozoa) replaced its ancestral chloroplast symbiogenetically by another from an undigested eaten haptophyte chromist (Patron *et al*. 2006). Independently, another small dinoflagellate lineage replaced its plastid by one from a green alga (Viridiplantae; dashed green arrow 1). Green algal chloroplasts were similarly independently implanted into Cercozoa (to make chlorarachnean algae; arrow 2) and into Euglenozoa (to make euglenoid algae; arrow 3). Euglenozoa, a phylum of ancestrally gliding zooflagellates (euglenoids; kinetoplastids, e.g. *Trypanosoma* and *Bodo*; postgaardiids; and diplonemids), differ so greatly from all other eukaryotes, and retain primitive bacteria-like features of mitochondrial protein-targeting and nuclear DNA pre-replication implying that they are the earliest diverging eukaryotic branch (Cavalier-Smith submitted *b*). Excavates comprise three entirely heterotrophic phyla: the putatively ancestral largely aerobic phylum Loukozoa (jakobids, which retain the most bacteria-like mitochondrial DNA, and *Malawimonas*), the largely aerobic derived phylum Percolozoa, and the secondarily anaerobic phylum Metamonada (e.g. *Giardia* and *Trichomonas*) that converted its mitochondria into hydrogenosomes or mitosomes and lost their genomes. Similar anaerobic relics of mitochondria evolved independently in Fungi, Amoebozoa, Percolozoa, Euglenozoa and Chromista. Contrary to earlier ideas, there are no primitively amitochondrial or primitively non-ciliate eukaryotes; earliest eukaryotes were aerobic flagellates, some of which evolved pseudopodia and became amoeboflagellates or eventually just amoebae. Animals and fungi both evolved from the same protozoan phylum, Choanozoa, but from different subgroups, being sisters of choanoflagellates and nucleariids, respectively (Shalchian-Tabrizi *et al*. 2008; Cavalier-Smith 2009*a*). Corticates and Eozoa are grouped as ‘bikonts’; formerly, the root of the eukaryote tree was postulated to be between unikonts and bikonts, not between Euglenozoa and excavates as shown here and justified in detail elsewhere (Cavalier-Smith submitted *b*)—a reassessment needing extensive testing.

- *An anaerobic phase in which photosynthetic non-sulphur bacteria* (*and before them extinct stem negibacteria*) *were the major primary producers for ecosystems*. The major consumers with surviving descendants were heterotrophic chlorobacteria, and perhaps also Hadobacteria if they preceded the origin of photosystem II. Exclusively anaerobic life probably persisted for roughly a billion years (from approx. 3.5 Gyr ago, the consensus but controversial date for the origin of life, to just under approx. 2.5 Gyr ago, the best date for the origin of photosystem II and oxygenic photosynthesis (Kirschvink & Kopp 2008). Claims for an earlier origin approx. 2.78 Gyr ago have been invalidated by the discovery that the hydrocarbon hopanoid biomarkers on which they were based (anyway not specific for cyanobacteria; Rashby *et al*. 2007) are not contemporaneous with the rocks whence they came (Rasmussen *et al*. 2008); they may be substantially younger (the same evidence invalidates claims using sterane biomarkers that eukaryotes are comparably old, always extremely discordant with the most conservative estimates based on morphological fossils of 800–850 Gyr (Cavalier-Smith 2002*a*); steranes are also not specific for eukaryotes). Though there have been claims for bacterial body fossils during this period, all are very nondescript (Schopf & Klein 1992); none is assignable to a particular phylum or subkingdom or even beyond reasonable question a genuine cellular fossil. Evidence for life is extremely indirect, mainly restricted to stromatolites (which could have been produced by filamentous Chlorobacteria or by stem bacteria now extinct) and isotopic signatures many of which may be genuinely biogenic but are prone to overoptimistic interpretations based on preconceptions and limited understanding of which aspects of presently known isotopic fractions can legitimately be extrapolated backwards into the Archaean, where we have no direct knowledge of which organisms were actually present. I call this the age of Eobacteria, though there is no direct evidence how far Chlorobacteria go back into this period or of when they replaced simpler stem bacteria that must once have existed.Though evidence for chlorobacteria being the most ancient cell type is good, I know none that can distinguish between their being sisters to all other organisms (as in figure 4) or a paraphyletic group ancestral to all other cells. Whichever is correct, if the tree is correctly rooted beside chlorobacteria as shown, or within them, they provide the best evidence we have for reconstructing the nature of the first cells. Aerobic life only evolved with the origin of oxygenic photosynthesis, which occurred at one of two points (figure 4): either immediately prior to the divergence of cyanobacteria and flagellate bacteria or somewhat earlier prior to the divergence of Hadobacteria.
- *The age of cyanobacteria* (*approx. 2.5–1.5 Gyr ago*) *in which cyanobacteria were the major primary producers and dominant morphological fossils*. However, very extensive anaerobic habitats probably remained, especially in the deep ocean. In the later part of this period, there are convincing body fossils of diverse cyanobacteria, including complex filamentous forms, some with heterocysts for fixing nitrogen (Schopf & Klein 1992). Major innovations during this period were: the origins of eubacterial flagella enabling life to move from ancestral benthic microbial mats into the plankton; the differential loss and modification of photosystem I or II to make three distinct phyla of anoxygenic phototrophs that could exploit anaerobic regions closed to cyanobacteria by acquiring novel antenna pigments enabling coexistence with and partial displacement of the more ancient chlorobacteria; and internalization of flagella to form spirochaetes able to corkscrew through soft anaerobic sediments. Concomitantly, there was massive metabolic diversification yielding a huge diversity of chemotrophic and heterotrophic negibacteria (especially by modifying the dominant purple bacteria, Proteobacteria) that greatly affected biogeochemical cycles.
- *The age of slowly increasing morphological complexity and colonization of continental surfaces by both Cyanobacteria and Posibacteria* (*1.5–0.85 Gyr ago*). In the past, some of the largest microfossils from this part of the middle Proterozoic have been attributed to eukaryotic algae; more recently many have been instead assigned to the fungi or (more plausibly in my view) to a mixture of complex Cyanobacteria and of the Posibacteria that display the greatest morphological complexity: the actinomycete Actinobacteria (Cavalier-Smith 2006*c*). Possibly therefore Posibacteria originated about 1.5 Gyr ago. No fossils in this period can be assigned with confidence to any eukaryote phyla and none in my view can assuredly be identified as eukaryotes. Some have been thought to be stem eukaryotes of undefined affinities, but all identifications of fossils in this period (even my own) merit scepticism except for those that are almost indubitably filamentous cyanobacteria of various groups.
- *The age of eukaryotes and obvious macroorganisms* (*850–800 Myr ago to the present*). Protozoa became the major predators on bacteria in water and wet earth; typically brownish photophagotrophic and photosynthetic chromists conquered the oceans; a green alga became a land plant 400 Myr ago, its descendants coating the continents where not too dry or cold with a green veneer providing homes and food for descendants of mobile animals (bilateria) that evolved 530 Myr ago via Cnidaria from marine sponges that fed on bacteria, like their choanoflagellate protozoan ancestors (figure 6). One choanoflagellate created sponges by evolving epithelia and connective tissue to allow more extensive filter feeding, and anisogamous sex to allow non-feeding ciliated larvae to grow large before settling onto rocks to feed. A distant choanozoan relative encased its filopodia in chitinous walls to evolve fungi that colonized soil as saprotrophs on dead plant material and symbionts and parasites of land plants. Archaebacterial sisters of eukaryotes colonized extreme habitats, one lineage evolving methanogenesis, changing climatic history by producing methane far faster than inorganic processes and triggering evolution of methanotrophs, mostly eubacteria (Cavalier-Smith 2006*a*); some methanogens invaded animal guts, evolving novel pseudomurein walls to evade digestion by host proteases.

The extremely complex origin of the eukaryotic cell initiated the modern world (phase 4) only after over three-quarters of the history of life was already over. This late origin of eukaryotes is attributable to the lateness of the immediately preceding neomuran revolution, during which bacterial secretion mechanisms and cell wall chemistry radically changed, allowing for the first time enough cell surface flexibility for evolution of phagocytosis of other cells. The inherent difficulty and improbability of the neomuran revolution, rather than the succeeding changes that made eukaryotes, probably accounts for the lateness of their origin and that of brainy life during the Cambrian explosion as a result of the origin of the anus and continuous flow processing of food (Cavalier-Smith 2006*a*).

It is no longer phylogenetically acceptable to assume that methanogenic archaebacteria existed in the Archaean age of anaerobic life and that their biogenic methane saved the Archaean world from global freezing. Either there was a now-extinct group of negibacteria that could make methane or, more likely, a mixture of carbon dioxide, water vapour and abiogenic methane were the major greenhouse gases maintaining climatic stability. Oxidative removal of abiogenic methane by the origin of oxygenic photosynthesis approximately 2.5 Gyr ago probably precipitated the Palaeoproterozoic global freezing approximately 2.3–2.4 Gyr ago (Kirschvink *et al*. 2000; Kopp *et al*. 2005; Kirschvink & Kopp 2008). Conversely, explosive production of methane by archaebacterial methanogenesis, significantly after neomura originated roughly 850 Myr ago, arguably destabilized climates by sudden runaway global warming and a reverberating intense cooling, inducing Neoproterozoic snowball Earth episodes (Cavalier-Smith 2006*a*).

The idea of Weismann and Wallace that asexual non-recombining organisms cannot evolve is wrong. Evolution in phases 1–3 was clonal: asexual cell lineages diverged without ever fusing. Some gene exchange occurred by viruses and in some groups by infectious plasmids or incidentally via food DNA (genetic transformation; Redfield *et al*. 2006). But recombination was not fundamentally important for evolution; it evolved primarily for DNA repair to stop harmful change. LGT was an incidental consequence of this; effects were often neutral though sometimes of adaptive significance, as in the evolution of eubacterial thermophily, drug resistance, host range or acquisition of foreign enzymes. But progressive changes in basic cell structure and the initial evolution of each metabolic pathway probably depended largely on mutation and vertical inheritance. Sex probably originated relatively late during eukaryogenesis, as a consequence, not cause, of the preceding changes in cell structure (Cavalier-Smith 2002*b*). Recombination is probably more important for the preservation of complexity than for its origin.

## 10. The cambrian explosion and early eukaryote phylogeny

The Cambrian explosion of novel animal phyla was immediately preceded by and overlapped with a similar explosion of protozoan and eukaryote algal phyla. This close timing of protist and animal megadiversification is most simply interpreted as the natural biological outcome of the somewhat earlier origin of phagotrophy and the eukaryotic cell itself, before which neither animals nor the enslavement of cyanobacteria to form eukaryotic algae and belatedly land plants was possible (Cavalier-Smith 2006*a*). Figure 6 summarizes deep eukaryote phylogeny, showing that the animal and fungal kingdoms both evolved from choanozoan ancestors and that origins of the plant and chromist kingdoms lie in the other half of the protozoan tree. After the origin of the eukaryote cell, few major innovations in cell structure were needed before these four derived kingdoms could have evolved (see Cavalier-Smith (2009*a*) for details on early eukaryote body plans and Cavalier-Smith (submitted *b*) for deep eukaryotic phylogeny). Although eukaryotes originated at least by 800 Myr ago, the period 800–600 Myr ago was considerably occupied by roughly three successive near-global glaciations (snowball Earth), which surely would have retarded early protist diversification. It cannot be coincidental that the largest expansion of protist diversity in Earth history immediately followed these global glaciations. The pump was primed by the earlier origin of eukaryotes. Glacial melting did not initiate cellular innovation; it just released the pent-up potential for innovation and rapid radiation that major new body plans themselves create. However, the symbiogenetic origin of chloroplasts may have taken place only about 600 Myr ago, immediately after the global snowball unfroze (most probably from 850 to 600 Myr ago the early eukaryotic photosynthesizers lived only by temporarily harbouring unintegrated cyanobacteria in their cells, as ‘pseudoalgae’ analogous to corals and green hydra, though eukaryotic algae might also have evolved earlier and failed to survive the freezing). Had archaebacteria never evolved and Neoproterozoic snowball Earth never occurred, the Cambrian explosion could have occurred 100 Myr earlier. But if the eubacterial cell wall necessarily prevented evolution of phagocytosis, phagocytosis could not have preceded the neomuran revolution and had to wait billions of years until that enabling change in wall chemistry.

## 11. Stasis, consTructional constraints and the rarity of mega-evolutionary innovation

The actual steps by which individuals come to differ from their parents are due to causes other than selection, and in consequence, evolution can only follow certain paths. These paths are determined by factors which we can only very dimly conjecture.

(Haldane 1932, pp. 142–143)

Variations are not, as Darwin thought, in every direction … Mutations only seem to occur along certain lines, which are very similar in closely related species, but differ in more distant species.

(Haldane 1932, p. 139)

Contrary to what I implied above, purifying/stabilizing selection is not the sole cause of stasis. Constructional constraints that make some phenotypes much more readily mutable than others are often equally important. The extreme stability over 3.5 Gyr of the negibacterial body plan with two bounding membranes, compared with unimembrana with one, is not explicable by harmfulness of mutations changing it but by their extreme rarity. The OM arguably evolved and was lost only once in the history of life (Cavalier-Smith 1987*a*, 2006*c*); it is exceedingly difficult to see how any DNA mutation could eliminate the OM except by the mechanism proposed for the origin of unibacteria (Cavalier-Smith 1980, 2006*c*): murein hypertrophy preventing insertion of lipid and proteins synthesized elsewhere in the cell into it. Such a mechanism is unavailable to mitochondria or most plastids, which are therefore irretrievably encumbered with a double envelope, irrespective of whether they would in principle function better and more efficiently without them. It is unreasonable to argue that having two membranes around a plastid is adaptive or optimal, still less for the optimality of having four around them as most chromists do (figures 1 and 6) simply because of an accident in history impossible to reverse or substantially improve upon. There is no reason whatever to think that the basically different body plan of photosynthetic chromists compared with plants (i.e. with plastids inside a periplastid membrane) is functionally an improvement; it is probably simply irreversible because no DNA mutation is possible that would remove three theoretically unnecessary membranes and relocate needed functions in just one. The complex lipid- and protein-insertion machinery is geared to retain the status quo and is permanently locked in complexity just as were the origins of the endomembrane system during the origin of eukaryotes, for which mutational reversal is inconceivable. The convergently evolved three membranes bounding dinoflagellate and euglenoid plastids are similarly frozen accidents (Cavalier-Smith 2003), like the specific details of the genetic code, and not adaptive.

Thus, progressive evolution is not inexorable, as Lamarck supposed, but has fits and starts, some especially significant for dividing the continuous tree of life into discrete taxa with radically different phenotypes durable over many hundreds of millions of years without radical evolutionary change. Lamarck imagined a polyphyletic origin of life, with inevitable steady upward progress; he supposed that unicellular organisms such as bacteria simply originated much more recently than groups such as vertebrates and therefore had less time to evolve greater complexity. That view of steady change is wrong. Bacteria have been around far longer, but failed (except when one lineage became the first eukaryote) to evolve greater complexity despite mutations in every part of every gene in every generation for over three billion years, roughly a trillion generations—such is the power of constructional constraints and stabilizing selection to prevent radical evolutionary change. They enable ancestral (paraphyletic) groups to retain phenotypic coherence and validity as taxa, despite the occasional relatively rare origin from them of new body plans, themselves mostly stable for hundreds of millions of years. By my counting, fewer than 60 phyla evolved in the history of life (Cavalier-Smith 1998). Probably none and very few class-level body plans ever became extinct and few if any major adaptive zones were ever totally emptied by extinction throughout Earth history.

Yet the false Lamarckian view of a steady rate of evolution remains remarkably pervasive 150 years after Darwin wrote *The origin*, substituted the divergent tree model for linear progress, and argued that major new adaptive types could originate and radiate extremely rapidly compared with the generality of evolutionary change. Examples of touching faith in the uniformity of evolutionary rates include the false supposition that cryptomonads independently enslaved a red alga much more recently than other chromists, because they alone retain the red algal nucleus as a nucleomorph (Whatley *et al*. 1979); the false claim that rRNA is a molecular chronometer (Woese 1987); excessive respect for the myth of a biological sequence clock; the idea that we can infer antiquity independently of direct fossil evidence from the degree of genetic or phenotypic change; the idea that sister groups necessarily deserve equal rank (Hennig 1966); and the idea that older groups necessarily deserve higher ranks. Twenty-first-century biology deserves better than these pre-Darwinian hangovers. Taxonomic rank should reflect the magnitude of the phenotypic innovations that created the group's cenancestor, not cladistic or temporal properties of the tree, as Darwin, an excellent taxonomist, recognized. However, though noting the reality of stasis, Darwin overlooked the centrality of body-plan stasis in evolutionary explanations of the taxonomic hierarchy.

Thus, there can be an essential irreversibility of many innovations in body plan, enabling a minority of lineages to grow periodically more complex by successive steps (figures 1, 3, 4, 6). The eighteenth-century ladder of life was mistaken in its lack of branching, but not in representing genuine evolutionary progress. Lamarck was the first to realize that there is not a single ladder of life but several divergent or parallel ones, but unlike some later writers did not throw its progressive features out with the bathwater. As one of the very few systematists in the history of biology (a name he invented) to work successfully on the higher classification of both the animal and plant kingdoms, Lamarck saw an aspect of the big picture of evolution that Darwin and Wallace with their emphasis on adaptation and biogeography largely missed. This is the contrast that Lamarck drew between adaptive change through new habits and new environments and the inherent tendency of life to become more complex that is the dominant factor in evolving new body plans, which persist millions of years beyond any local selective forces that initiated them. Though (like both Darwins) Lamarck failed to understand that effects of changed habits on evolution were mediated by mutation and selection (Wallace (1889) and Weismann (1889) independently thus explained the evolutionary effects of ‘use and disuse’), he had a more balanced view than many of the interplay of internal and external factors in evolution and suffered unduly from misrepresentation of his actual views. As others like Whyte (1965) later emphasized, internal organismal factors in evolution must not be ignored. Wallace apparently never tried to explain the taxonomic hierarchy or published any tree, but took refuge in spiritualism and the idea of benevolent mind—helped by sundry subsidiary spirits—subtly diverting evolution away from its spontaneous tendencies towards usefulness for its crowning glory, civilized man (Wallace 1911); he rejected the pure mechanism of Maupertuis, Lamarck, Darwin and Haeckel as atheistic. Wallace (1911) thought that the origin of the eukaryotic cell required a designing mind; a mechanistic explanation now exists (Cavalier-Smith 2009*b*). Increases in cellular and organismal complexity do not require a guiding mind, but are inevitable eventual consequences of life only being able to start very simply (for a model starting with only three genes in our last common ancestor, see Cavalier-Smith 2001). Once life began, radiation in every direction allowed by existing constructional constraints and continued viability must inevitably increase complexity in some lineages, irrespective of equally inevitable secondary simplifications in others; how both occur is constrained not just by population genetics and ecology, but still more fundamentally by physical interactions and coevolution of different parts of the cell (Cavalier-Smith 2002*a*, 2006*a*, 2009*b*, in press), by developmental constraints in multicellular organisms (Raff 1996; Roux & Robinson-Rechavi 2008), and by the starting material available in each era from past phylogeny (phylogenetic constraints).

Historical accidents (e.g. which of several possibilities happened first) can become fixed as phylogenetic constraints. Thus, adaptations for phagotrophy almost certainly played a key role in initiating eukaryogenesis, but the endomembrane system, cytoskeleton and mitosis that evolved as a result of historical accidents and the inner logic of recovery from the associated disruptions (Cavalier-Smith 2009*b*) persist unchanged in plants, fungi and others that have long since given up phagotrophy simply because of constructional inertia and the irreversibility of complex evolution. Likewise, adaptedness to hyperthermophily probably favoured the origin of novel archaebacterial lipids, but played no role in their retention by secondary mesophiles, which was just constructional inertia coupled with the impossibility of re-evolving the old type or regaining them by LGT. The loss of the negibacterial OM may never have been directly selected at all, but was an indirect mechanistic consequence of murein hypertrophy that might itself have been an adaptation against desiccation (Cavalier-Smith 1980). Such constructional complications, what Darwin (1859) called ‘mysterious laws of the correlation of growth’—the sphere of cell and developmental biologists—are very important for evolutionary biology, yet outside the scope of the population genetics approach to evolution, which though illuminating is necessarily limited through sidestepping the specifics of actual phenotypes, particular phylogenies and unique historical accidents.

As always, Haldane (1932, pp. 104–105), the prime mover of modern evolutionary theory, was ahead of the pack in recognizing a role for constructional constraints in channelling large-scale evolution and in accepting that when ‘a successful evolutionary step rendered a new type of organism possible’, major subgroups arise relatively suddenly ‘in an orgy of variation’ and that subsequent evolution is ‘a slower affair’. Darwin (1859) said it as strongly. Of the ‘new synthesis’ authors only Simpson (1944), who coined the term ‘quantum evolution’ for the ultra-rapid origin of a new body plan, fully appreciated the extreme rapidity of mega-evolution, another neglected Simpsonian concept that I seek to revive. My life-time studies of microbial evolution fully confirm Simpson's conclusions from animal palaeontology and highlight the fundamental misinterpretations of the tree of life that arose from the contrasting false belief in uniformism throughout phylogenetic history (Cavalier-Smith 2006*a*–*c*, 2009*a*,*b*, in press).

## 12. Need to intensify study of chlorobacteria

According to my recent analyses, Chlorobacteria are the most primitive extant cells (Cavalier-Smith 2006*a*,*c*). The misconception that Archaebacteria are extremely ancient early diverging cells especially significant for the origin of life (Woese & Fox 1977) has proved to be false (Cavalier-Smith 2006*a*,*c*). Widespread belief that it was true caused numerous fundamental misinterpretations of the tree of life and the dogmatism often associated with it has impeded more balanced understanding. However, faith in this fundamentally mistaken idea has also immensely stimulated research into archaebacteria for three decades, which has yielded innumerable valuable new discoveries and insights into microbiology. Moreover, as archaebacteria have turned out to be sisters of eukaryotes, the new facts were very important and beneficial for understanding their origin (Cavalier-Smith 1987*a*,*b*, 2002*b*, 2009*b*), though seeing archaebacteria as ancestral and ancient has been harmfully confusing and grossly misleading as to the nature of the last common ancestor of all life. Thus, intense recent archaebacterial research has been extremely productive and valuable, despite being totally irrelevant to and a distraction from understanding the origin of life. Better understanding of earliest evolution requires a comparable large-scale effort to elucidate the diversity, cell biology, and ecology of Chlorobacteria. If I am right about their deep phylogenetic position, this will greatly clarify the nature of the last common ancestor of all life. Even were I wrong, such research would hugely advance understanding of an important, highly divergent bacterial phylum; probably the least understood of all 10 bacterial phyla that I currently recognize. Environmental DNA sequencing reveals numerous chlorobacterial lineages that have never been cultured. Only four genomes are sequenced (e.g. Seshadri *et al*. 2005; Wu *et al*. 2009) and the physiology and phenotypes of the vast majority of lineages are unknown. Chlorobacterial research is also important for biotechnology and bioremediation, as many (e.g. *Dehalococcoides*) anaerobically respire chlorinated hydrocarbons as food, playing a crucial role in their natural detoxification (Kittelmann & Friedrich 2008); might other novel metabolisms be revealed? Sceptics who wish to disprove my conclusions also should study chlorobacterial molecular and cell physiology to show how their cell envelopes work and see if they can explain how their apparently primitive properties might have evolved secondarily from other bacteria that I consider more advanced.

Membrane chemistry differs in the non-photosynthetic chlorobacterium *Thermomicrobium* from other negibacterial phyla by lacking glycerophospholipids (Wu *et al*. 2009) and having instead glycolipids based on long-chain diols (Pond *et al*. 1986; Wait *et al*. 1997), probably also present in the photosynthesic *Chloroflexus* (Woese 1987), and unusual glycosylated carotenoids (Wu *et al*. 2009); conceivably, these unusual lipids may stabilize chlorobacterial membranes in the absence of lipopolysaccharide or hopanoids. In addition to similar diol glycolipids, the hadobacterium *Thermus* possesses both phospholipids and glyceroglycolipids (Wait *et al*. 1997); this suggests that, if phospholipids and/or glycerolipids prove to be absent from all Chlorobacteria, one or both may have evolved after the divergence of hadobacteria and glycobacteria from them (figure 4). A phylogenetically broad survey of lipid chemistry and membrane organization (both the cytoplasmic and OM; how greatly do they differ?) among Chlorobacteria would test this and be important for correctly deducing the nature of the membranes in the last common ancestor of all life; contrary to widespread assumptions, such an ancestor might not have had any kind of phospholipid (whether the acyl ester phospholipids of non-chlorobacterial eubacteria or the isoprenoid ethers of archaebacteria) in its membranes; it might instead have had acyl ester diol glycerolipids, only later replaced in most organisms by glycerophospholipids, with hadobacteria an intermediate stage possessing both. Many cherished assumptions about early cellular evolution might be overturned by more thorough and phylogenetically representative study of the molecular cell biology of Eobacteria, including the many still uncultured chlorobacterial lineages known only from environmental DNA sequencing.

## Endnote

1 His letters published in this journal in its early days, by revealing the microbial world, led eventually to revision of the contemporary idea (Lemery 1675) of only three kingdoms of nature: animals, vegetables and minerals.
